# A Nanostructured Cu(II) Coordination Polymer Based on Alanine as a Trifunctional Mimic Enzyme and Efficient Composite in the Detection of Sphingobacteria

**DOI:** 10.1155/2022/8788221

**Published:** 2022-04-11

**Authors:** Noelia Maldonado, Ana Latorre, Félix Zamora, Álvaro Somoza, Carlos J. Gómez-García, Agatha Bastida, Pilar Amo-Ochoa

**Affiliations:** ^1^Departamento de Química Inorgánica, Universidad Autónoma de Madrid, Madrid 28049, Spain; ^2^Instituto Madrileño de Estudios Avanzados en Nanociencia (IMDEA Nanociencia), Cantoblanco, Madrid 28049, Spain; ^3^Institute for Advanced Research in Chemical Sciences (IAdChem), Universidad Autónoma de Madrid, Madrid 28049, Spain; ^4^Departamento de Química Inorgánica, Universidad de Valencia, C/Dr. Moliner 50 46100 Burjasot, Valencia, Spain; ^5^Departamento de Química Bio-Orgánica, Instituto de Química Orgánica General del CSIC, Madrid 28006, Spain

## Abstract

This research raises the potential use of coordination polymers as new useful materials in two essential research fields, allowing the obtaining of a new multiartificial enzyme with the capacity to inhibit the growth of bacteria resistance. The fine selection of the ligands allows the design of a new 2D coordination polymer (CP), with the formula [Cu_2_(IBA)_2_(OH_2_)_4_]_n_·6nH_2_O, by the combination of Cu (II) as the metal center with a pseudoamino acid (H_2_IBA = isophthaloyl bis *β*-alanine). Quantitative total X-ray fluorescence (TXRF) analyses show that the obtained CP can gradually release Cu (II) ions. Additionally, this CP can be nanoprocessed and transformed into a metal-organic gel (MOG) by using different Cu (II) salt concentrations and the application of ultrasounds. Considering its nanometric dimensions, the slow Cu (II) release and its simple processability, its performance as an artificial enzyme, and its antibacterial ability were explored. The results obtained show the first nanocoordination polymer acting as an artificial multienzyme (peroxidase, catalase, and superoxodismutase) exhibiting antibacterial activity in the presence of hydrogen peroxide, with selective behavior for three bacterium strains (*S. spiritovirum, A. faecales, and B. cereus*). Indeed, this CP shows a more robust inhibition capacity for *Sphingobacterium*. Going beyond that, as there are no comfortable and practically clinical tests capable of detecting the presence of *Sphingobacteria*, the compound can be easily embedded to form moldable gelatin that will facilitate the handling and low-cost commercial kits.

## 1. Introduction

Pathogenic intracellular bacteria have been proven to trigger numerous chronic or recurrent infectious diseases that pose substantial global public health threats. The main medical route for treating bacteria is antibiotics, but these microorganisms use many mechanisms to survive by becoming resistant [1]. For this reason, the discovery and development of alternative antimicrobial strategies are also critical [2–6]. Additionally, in the last decades, researchers have tried intensively to find new compounds capable of behaving as artificial enzymes, copying the functions of natural enzymes [7]. These artificial enzymes will be able to produce essential chemicals on an industrial scale with a performance that rivals their natural counterparts. Moreover, if we are inspired by the use that the human body makes of enzymes to improve its defense mechanisms, see, for example, the xanthine oxidase that can generate superoxide anion (O_2_^−^) and H_2_O_2_ in the presence of O_2_, or the myeloperoxidase that can catalyze the conversion of H_2_O_2_ into highly reactive oxygen species (ROS) that behave as natural antimicrobials, then artificial enzymes could also be used as alternative compounds in the fight against bacteria [8]. Investigations into these two subjects cover an immense variety of materials [9, 10]. However, coordination polymers can be a possible solution to both aspects since they have fascinating characteristics and properties to achieve these objectives. From the chemical point of view, a recently published precedent shows how coordination polymers (CPs) can be highly interesting in manufacturing mimic enzymes with, at the same time, antimicrobial capacity as they can generate ROS in a controlled way [11]. Indeed, one exciting advantage of the CPs as possible artificial enzymes is their facile preparation, nanoprocessability, low cost, and superior stability [12]. Other advantages of the CPs are their tuneability, since the proper selection of the building blocks (metal ions and organic ligand/s) should allow the obtaining of a material with the desired properties [13–16]. Thus, the use of copper (II) as a metal center enables the creation of compounds with different antibacterial mechanisms [17–19]. One can be related to the metal ion release in a controlled manner, favoring obtaining compounds that improve wound healing without generating toxicity. The metal ion release is related to the electrostatic attraction between the positively charged Cu(II) or Cu(I) ions and microorganisms negatively charged cell membranes, resulting in bacterial cell death by damaging the cell wall and plasma membrane [17]. Indeed, considering this type of strategy, A. Lauf et al. have proved that the copper (II) release rate is crucial in antibacterial effectiveness, finding that this efficacy can be improved as the particle size is reduced [20, 21]. Indeed, considering particle insertion into bacteria as a possible antibacterial mechanism, it has been demonstrated that Cu(II) coordination polymer nanofibers are more effective than the same compound in the form of microcrystals [17]. These research studies show that the synthesis of new Cu(II)-based structures with control in the Cu (II) ion release rate is essential [11, 17, 19, 22]. Additionally, the presence of this ion is critical to provide peroxidase [23], catalase, or superoxodismutase (SOD)-like activity. Organic ligands are also crucial to obtaining compounds of biological interest that when prepared with the appropriate synthetic conditions can generate metal-organic gels (MOGs) with three-dimensional network structures capable of harboring solvents. These new phases have been highly interesting for real industrial applications since they facilitate the localized superficial administration of metal ions as Cu (II) in the antibacterial treatment of external wounds [24, 25]. Other studies show that creating new composite materials by a combination of Cu (II) CPs and organic matrices such as polyacrylamide, cellulose, fiber, gelatin [26], chitosan (CS), or cotton [27–30], among others [31], provides improvements in antibacterial effectiveness in addition to new mechanical properties allowing their manufacture.

Despite these important discoveries, there are just a few examples of CPs combining multiple enzymatic actions, although none acting as a trifunctional mimic enzyme [32–34]. Here we present a study that combines Cu (II) with a pseudoamino acid (H_2_IBA = isophthaloyl bis *β*-alanine). The synthesis in a single step and sustainable conditions (water and 25°C) allows obtaining a 2D CP that can be nanoprocessed and gelled by sonication of different Cu (II) metal salts. The obtained CP, with the chemical formula [Cu_2_(IBA)_2_(OH_2_)_4_]_n_·6nH_2_O shows slow Cu (II) release and is the first example of a trimimic artificial enzyme having peroxidase, catalase, and superoxodismutase like activity, being effective for the resistant *Sphingobacterium.* In addition, the easy preparation of the corresponding metal-organic gel and also a composite material based on gelatin and the nanocoordination polymer (NCP) will allow the creation of easy-handling and low-cost commercial kits valid for detecting *Sphingobacterium* [35, 36].

## 2. Results and Discussion

### 2.1. Chemical and Morphological Characteristics of **1n**

**Figure 1:**
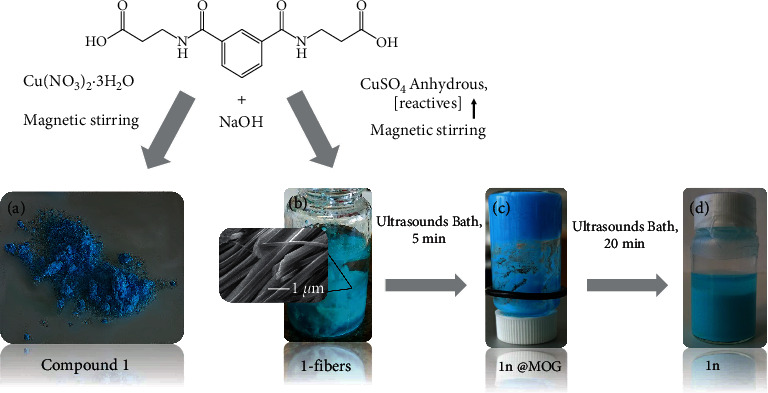
Different conditions to obtain compound **1** as bulk material (a), fibers, 1-fibers (b), metal-organic gel, **1n**@MOG (c), or nanocrystals, **1n** (d).

An essential advantage in the synthesis of this CP is the possibility of obtaining it in different phases and sizes by slightly modifying the synthetic conditions. That is, the direct reaction between H_2_IBA deprotonated with NaOH and Cu(II) can generate compound **1** as single microcrystals, as polycrystalline, as a colloid formed by nanofibers (**1n**), or as metal-organic gel (**1n**@MOG) depending on the starting copper (II) salt (Cu(NO_3_)_2_ or CuSO_4_) and the application of different sonication times, as shown in Figure 1 (methods described in Supplementary Materials) [37]. The characterization of **1**, **1n**, and **1n**@gelatin by IR (Figure S1), PXRD (Figures S2 and S3), and SEM (Figure S4) has been carried out.

**Figure 2:**
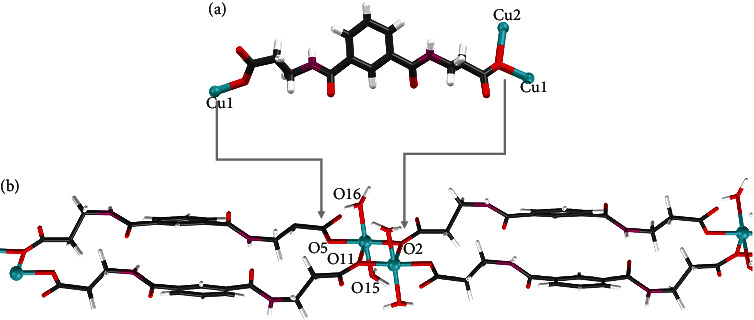
Isophthaloyl bis *β*-alanine (H_2_IBA) coordination modes to Cu (II) metal centers in compound **1** (a). Structure fragment of compound **1** (b). Color code: Cu = light blue, O = red, N = purple, C = grey, and H = white.

Structurally speaking, compound **1** is a 2D coordination polymer that crystallizes in the monoclinic *Pc* space group [38]. Its structure has been described previously by S. Lymperopoulou et al. which consists of Cu(II) dimers where both metal ions present a distorted square pyramidal coordination geometry (Figure 2). The basal plane of both Cu (II) ions is formed by two water molecules (with Cu-O bond distances in the range 1.934–1.983 Å) and by two *trans* carboxylate oxygen atoms from two different IBA^2−^ ligands: a terminal one, with Cu-O bond distances of 1.926(3) and 1.922(3) Å, and a bridging one, with Cu–O bond distances of 1.961(3) and 1.942(3) Å, for Cu_1_ and Cu_2_, respectively. The apical position is occupied by the bridging carboxylate oxygen atom of the basal plane of the other Cu (II) ion, giving rise to a central CuO_2_Cu dimer. The apical Cu–O bond distances (2.366(3) and 2.402(3) Å) are much longer than the basal ones.

**Figure 3:**
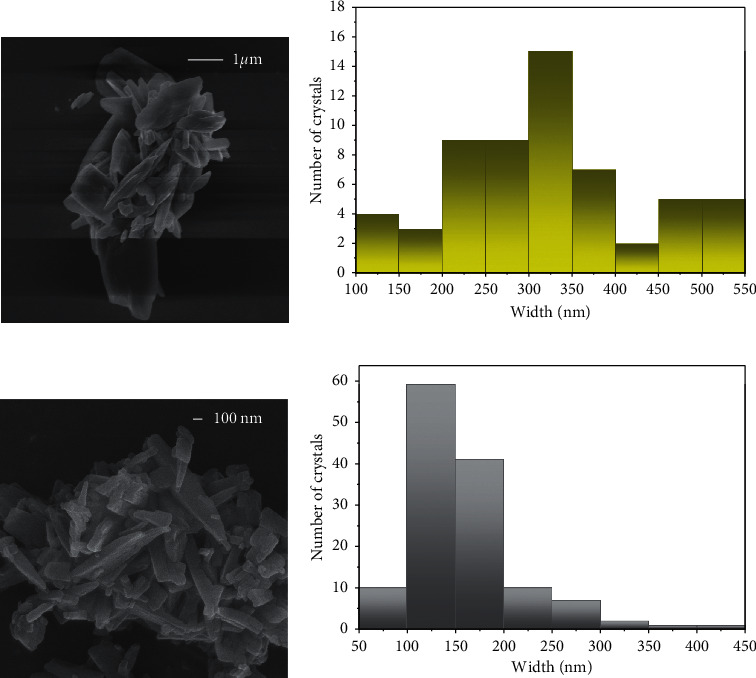
SEM images of compound 1 (a) and 1n (c) together with statistical histograms of widths (b, d).

**Figure 4:**
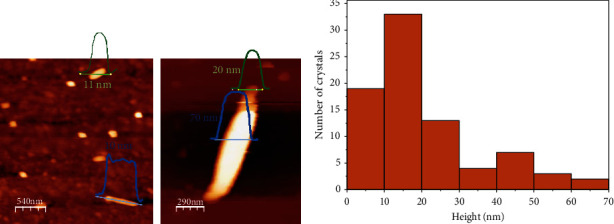
AFM images of compound **1n** (a) and statistical histogram of height (b).

As ultrasounds are used to assist sol-gel transitions [39, 40], a bottom-up approach using sonication and CuSO_4_ instead of Cu(NO_3_)_2_ results in the formation of a metal-organic gel (**1n@MOG**) which is transformed into a colloid formed by the corresponding nanocrystals (**1n**) with longer sonication times (Figure 1 (c) and (d)). As shown in Figure S4, the use of CuSO_4_ instead of Cu(NO_3_)_2_ seems to enhance the reduction of particle size. The morphology and dimensions of both **1** and **1n** were studied by SEM and AFM (Figures 3 and 4). The average width for 1 ribbons and **1n** nanoribbons is 354 ± 170 nm and 170 ± 60 nm, respectively, while the average height for **1n** is 17 ± 10 nm.

Compound **1n** is stable at physiological pHs (between pHs 3 and 8) for long periods of time (one year) (Figures S6 and S7). Additionally, Figure S8 shows thermal stability up to 254°C. It loses all the solvation water molecules at this temperature, and the coordinated ligands decompose mainly into CO_2_. At temperatures above 100°C, the blue color of compound **1n** reversibly transforms into a new less crystalline green color phase due to the loss of the first solvated water molecules (Figures S9–S11). The magnetic properties of **1n** show the presence of a metamagnetic behavior with a high critical field of *ca*. 5.5 T at 2 K, i. e., the Cu (II) dimer shows a weak antiferromagnetic coupling of −6.0 cm^−1^ that becomes ferromagnetic for applied magnetic fields above 5.5 T, as depicted in Figures S12 and S13.

### 2.2. Catalase Mimicking Activity of **1n**

**Figure 5:**
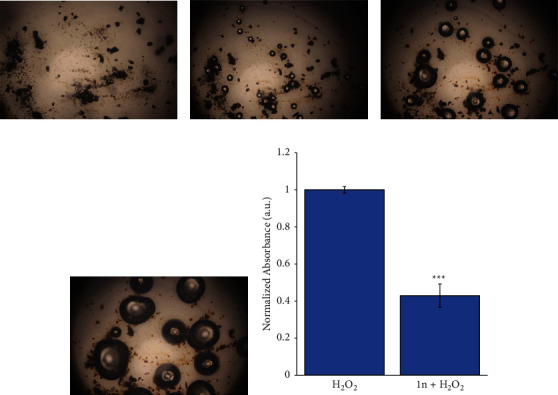
Oxygen bubbles generation when **1n** is exposed to H_2_O_2_ 30% for (a) *t* = 0, (b) *t* = 10 s, (c) *t* = 20 s, and (d) *t* = 30 s. Absorbance of chromic acetate in the presence of H_2_O_2_ (left bar) and after the incubation with **1n** (right bar). The data correspond to mean ± S.D. values from four experiments (e). Statistical analysis was performed using one-way ANOVA Tukey's test (each group vs. control). ^*∗∗∗*^*P* < 0.0001.

Catalase is responsible for the catalytic decomposition of hydrogen peroxide through its disproportionation reaction into nontoxic dioxygen and water. The catalytic activity of **1n** (methods in Supplementary Materials) [37] towards the decomposition of hydrogen peroxide has been investigated in water at 25°C. Initially, the suspension of **1n** is dark blue, but after adding 30% (v/v) hydrogen peroxide, it became faded yellow and rapid evolution of gas was observed, such as in Figures 5(a)–5(d). It can be easily understood that the evolved gas is oxygen, and it comes from the catalytic decomposition of hydrogen peroxide solution [41].

Moreover, to quantitatively estimate the catalase activity of compound **1n**, a colorimetric assay was performed by titration of excess unreacted H_2_O_2_. In this method, the decomposition of H_2_O_2_ is estimated spectrophotometrically by a reaction with potassium dichromate/acetic acid reagent [42] (catalytic activity: methods in the Supplementary Materials). In the presence of H_2_O_2_, potassium dichromate in acetic acid is reduced to green-colored chromic acetate, which can be measured colorimetrically at 570 nm. As shown in Figure 5(e), when H_2_O_2_ is added to the **1n**, the absorbance signal decreases significantly from 1 to 0.43 after 2.5 hours of incubation. This result demonstrated that compound **1n** presents catalase-like activity, directly proportional to the dissociation rate of the H_2_O_2_ produced in the samples.

### 2.3. Peroxidase Mimicking Activity of **1n**

**Figure 6:**
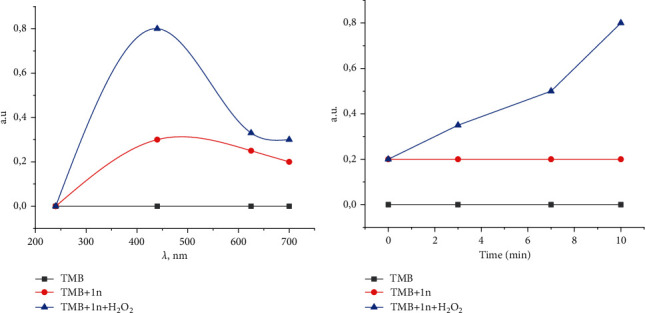
(a) Absorbance spectra from 200 to 700 nm of TMB (grey line), TMB + **1n** (red line), and TMB+**1n** + H_2_O_2_ (blue line). (b) Time-dependent absorbance changes at 440 nm of oxidized TMB (grey line), TMB + **1n** (red line), and TMB + **1n** + H_2_O_2_ (blue line).

The excellent intrinsic peroxidase-like activity of **1n** was evaluated by oxidizing chromogenic peroxidase substrate 3,3′,5,5′-tetramethylbenzidine (TMB) to form a blue-colored product under the assistance of H_2_O_2_ (catalytic activity: methods in Supplementary Materials) [37]. TMB itself is colourless and displays no absorbance (Figure 6) [43]. As depicted in Figure 6(a), a strong absorption was observed in the group of TMB **+** **1n** **+** H_2_O_2_, indicating that **1n** possesses an excellent catalytic activity. Moreover, a time-dependent change in the TMB absorbance was recorded by a UV–vis spectrophotometer at 440 nm (Figure 6(b)).

### 2.4. Superoxo-Dismutase (SOD) Mimicking Activity of **1n**

**Figure 7:**
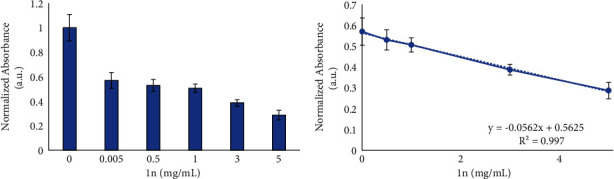
Decrease in NBT absorbance versus progressive increase in **1n** concentration (0–5 mg/mL) (a) and **1n** concentration that inhibits the NBT reduction rate by 50% (b). IC_50_ calculation using linear regression analysis, employing five different concentrations of **1n**. The data correspond to mean ± S.D. values from three experiments. Statistical analysis was performed using one-way ANOVA Tukey's test (each group vs. Control). ^*∗∗∗*^*P* < 0.0001.

To determine if **1n** can be a candidate as a mimic antioxidant enzyme useful, for example, in the treatment of disorders related to oxidative stress, we have studied its activity as a superoxodismutase (SOD) [34]. To evaluate this SOD-like activity, an assay with nitroblue tetrazolium (NBT) was performed to quantify the **1n** enzymatic activity (catalytic activity: methods in the Supplementary Materials; Figures S14 and S15) [37]. The reaction kinetics between NBT and the xanthine/xanthine oxidase (x/xo) system were measured to establish when the absorbance reaches its maximum value at a wavelength of 560 nm. Triplicate NBT spectra were then performed with different concentrations of **1n** in a stepwise manner (0, 0.005, 0.5, 1, 3, and 5 mg/mL), observing a continuous decrease of the signal as the concentration of **1n** increases, which disappears entirely at a concentration of 3 mg/mL. Nearly 80% of the superoxides were removed from a concentration of 5 mg/mL of **1n**, and IC_50_ = 1.1 mg/mL was calculated (Figure 7) to establish the concentration of **1n** that inhibits the rate of NBT reduction by 50%.

### 2.5. Antibacterial Assays

To determine the copper concentrations produced when compound **1n** is in suspension, release assays of the compound in deionized water were carried out (Figure S16). Considering the system's slow Cu(II) release (45 ppm at 24 h), its nanometric dimensions, synthesis in the water a room temperature in just one step, and its ability to act as a multiartificial enzyme [11], its antimicrobial activity [44] was also explored.

The antibacterial activity of **1n** compound and **1n** + H_2_O_2_ was tested by agar diffusion against different microorganisms (antibacterial experiments in Supplementary Materials) [37] including Gram-positive (*Bacillus*, *Deinococcus*, and *Lactococcu*s *lactis*) and Gram-negative (*Escherichia*, *Pseudomonas*, and *Sphingobacterium*, *Alcaligenes*). Furthermore, different concentrations of H_2_O_2_ alone were checked against *Sphingobacterium* to discard the growth inhibition by oxygen peroxide's effect at the range of work concentrations, only observing a slight halo at 300 mM, as shown in Figure S17.

As well, the minimal inhibitory concentration (MIC) and growth curves of the compound **1n** without and with H_2_O_2_ versus different strains were assayed (Table S1 and Figure S18).

The results obtained indicate that compound **1n** did not show growth inhibition against any strain, which means that **1n** is not an antimicrobial agent.

**Figure 8:**
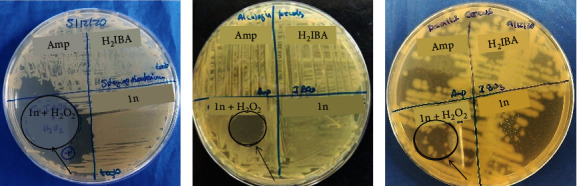
Antibacterial experiments photographs of compound **1n** + H_2_O_2_ (25 mM) where CuIB03 = **1n**; IB03 = H_2_IBA; Amp = ampicillin) tested on. *Sphingobacterium* (a), *Alcaligenes faecalis* (b), and *Bacillus cereus* (c). Ampicillin is used as antibiotic control.

But **1n** + H_2_O_2_ inhibits the growth of *A. faecalis, B. cereus (*to a lesser extend*), and Sphingobacterium,* probably due to the generation of reactive oxygen species [45–47] (Figure 8).

Most isolates from humans are *Sphingobacterium spiritivorum* which are generally resistant to kanamycin and ampicillin and are susceptible to the quinolones and trimethoprim-sulfamethoxazole.

Bacterial growth of *Sphingobacteria* was also measured by the optical density method at 600 nm (OD_600_) overnight at 37°C. After 24 h in the presence of **1n** + **H**_**2**_**O**_**2**,_ no bacterial growth was detected that performed as an antimicrobial agent (Table 1).

**Figure 9:**
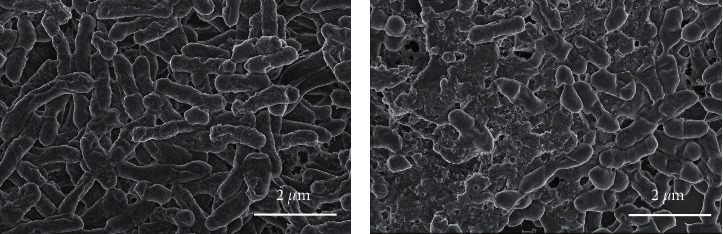
FESEM images of *Sphingobacterium* with no treatment (a) and after being treated with 100 *μ*L **1n** + 50 *μ*l H_2_O_2_ (30%) for 24 h(b).

Possible changes in *Sphingobacterium* morphology were explored using FESEM. In Figure 9, the damages caused by the presence of **1n** + H_2_O_2_ upon 24 h to the bacterial surface were clearly observed regarding the control, probably due to the generation of ROS (O_2_, HO^·^) that could oxidize the cell membranes as has been concluded using 3,3′,5,5′-tetramethylbenzidine (TMB) or catalase experiments [48].

### 2.6. Efficient Composite (**1n**@Gelatin) in the Detection of Sphingobacteria

**Figure 10:**
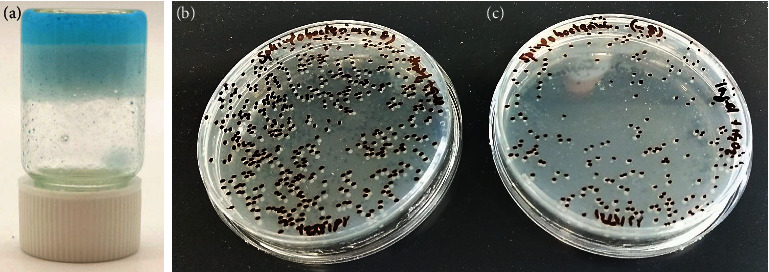
Photographs of **1n@Gelatin** (a) and *Sphingobacterium spiritivorum* colony count method (50 *μ*L of overnight of culture after 10^−8^ dilution) (b) where 490 colonies were counted for the blank experiment (40 *µ*L of H_2_O_2_ at 25 mM) and (c) 210 colonies were counted after being treated *Sphingobacterium* with **1n@Gelatin** (40 *µ*L at 3 mg/mL) plus H_2_O_2_ (40 *µ*L at 25 mM).

The prepared metal-organic gel of **1n** (**1n**@MOG) was not stable at physiological pH (only around pH 3). Therefore, we have used gelatin as a biocompatible organic matrix in order to process **1n** in a moldable and easy way creating a gel-like composite (Figure 10(a)). This new composite allows the release and diffusion of **1n** during the incubation stage thanks to its excellent solubility in water at 37°C. With compound **1n** in this format, we have achieved an improved manufacture and handling of the material, facilitating its application *in vitro* tests compared to bulk material.

The antibacterial efficacy of **1n@Gelatin** was checked against *Sphingobacterium* by the colony count method (antibacterial experiments in Supplementary Materials) [37]. The number of grown colonies of *Sphingobacterium* overnight at 37°C was 490 colonies, while in the presence of **1n**@Gelatin plus H_2_O_2_ (25 mM) it was half (Figure 10(b)). From these results, the percentage of inhibition (K inhibition (%)) of **1n**@Gelatin was evaluated versus *Sphingobacterium* and *E*. *coli* DH5*α*. The results showed *K* = 57% for *Sphingobacterium* inhibition, while there was no decrease in the number of colonies for *E*. *coli* DH5*α*.

## 3. Conclusions

This research raises the potential use of coordination polymers as new useful materials in two significant fields of research, allowing the obtaining of new artificial enzymes with the capacity of inhibiting the growth of bacteria resistant to the usual antibiotics. The adequate selection of the ligands has enabled the design of the first artificial trienzyme model (peroxidase, catalase, and superoxodismutase) based on a Cu (II) CP with modified alanine. It is worth emphasizing that studies based on the search for enzyme mimics are focused on organic materials, but the study of artificial enzymes with CPs is practically nonexistent, despite the good characteristics that are present in CPs based on biologically relevant ligands. Remarkably, its excellent catalytic activity in the presence of minimal amounts of hydrogen peroxide is essential for its antibacterial effect on three resistant bacteria strains. Another critical aspect of this work is that Gram negative bacteria (*Alcaligenes faecalis* and *Sphingobacterium*) are usually involved in respiratory infection processes and present a vast antibiotic resistance, making this CP a powerful alternative for its use against these types of pathogens. The damages caused to the bacterial wall indicate indirect evidence of the generation of oxidizing hydroxyl species or reactive oxygen species due to the CP plus minimal concentrations of H_2_O_2_. Finally, the transformation of the CP into a metal-organic gel (MOG) could facilitate its use in the manufacture of new commercial *Sphingobacteria* detection kits. However, the MOG obtained does not present enough stability at physiological pH. For this reason, as a proof of concept, this compound has been homogeneously dispersed in soluble gelatin as an easily useable and moldable organic matrix with excellent antibacterial results which will allow the manufacture of low-cost, comfortable, easy-to-use, and affordable commercial *Sphingobacteria* kits for assessing the presence of microorganisms in a test sample employing the identification of the inhibition of the microorganism.

## Figures and Tables

**Table 1:** OD values at 600 nm of the *Sphingobacterium* in the presence of kanamycin antibiotic, H_2_O_2_, **1n**, and **1n** plus H_2_O_2_.

Strain/compound	OD_600nm_, 24h 37°C
Sphingobacterium + **1n**	2.67
Sphingobacterium + Kanamycin	2.44
Sphingobacterium + H_2_O_2_	2.34
Sphingobacterium + CuSO_4_	1.35
Sphingobacterium + **1n** + H_2_O_2_	0.05

## Data Availability

The data underlying the findings of this paper are publicly available. All the obtained and used data are available and have been deposited in a public repository whose access link is: https://doi.org/10.21950/NR3KKL. Additionally, they are included in the article in the form of supplementary material (PDF file with the name: SI_bioinorg_chem_app_20_1_22).

## Supplementary Materials

Supplementary Materials S1 experimental section: materials and instrumentation; S2 synthesis of **1**, **1n**, and **1n**@Gelatin; S3 characterization of **1**, **1n**, and **1n**@Gelatin; S4 solvent and thermal stability of **1n**; S5 study of magnetic properties; S6 catalytic activity: methods; S7 copper release and antibacterial experiments; and S8 references. This material can be found in https://edatos.consorciomadrono.es/dataset.xhtml?persistentId=doi:10.21950/NR3KKL.
