# Supplementary material for: A Nanostructured Cu(II) Coordination Polymer Based on Alanine as a Trifunctional Mimic Enzyme and Efficient Composite in the Detection of Sphingobacteria
Source: Bioinorg Chem Appl. 2022 Apr 11;2022:8788221. doi: 10.1155/2022/8788221 (PMC9017554; doi:10.1155/2022/8788221)
Supplement: Supplementary Materials — S1 experimental section: materials and instrumentation; S2 synthesis of 1, 1n, and 1n@Gelatin; S3 characterization of 1, 1n, and 1n@Gelatin; S4 solvent and thermal stability of 1n; S5 study of magnetic properties; S6 catalytic activity: methods; S7 copper release and antibacterial experiments; and S8 references. This material can be found in https://edatos.consorciomadrono.es/dataset.xhtml?persistentId=doi:10.21950/NR3KKL. [file 8788221.f1.docx]

**Electronic Supplementary Material**

| **A nanostructured Cu(II) coordination polymer based on alanine as a tri-functional mimic enzyme and efficient composite in the detection of *Sphingobacteria*** |
| --- |
| Noelia Maldonado^1^, Ana Latorre^2^, Félix Zamora^1,5^, Álvaro Somoza^2^, Carlos J. Gómez-García^3^, Agatha Bastida^4^ (**🖂**), Pilar Amo-Ochoa^1,5^ (**🖂**)  *^1^ Departamento de Química Inorgánica, Universidad Autónoma de Madrid, 28049 Madrid, Spain*  *^2^ Instituto Madrileño de Estudios Avanzados en Nanociencia (IMDEA Nanociencia), Cantoblanco, 28049 Madrid, Spain*  *^3^ Departamento de Química Inorgánica, Universidad de Valencia, C/ Dr. Moliner 50, 46100 Burjasot. Valencia, Spain*  *^4^ Departamento de Química Bio-Orgánica. Instituto de Química Orgánica General del CSIC, 28006 Madrid, Spain*  *^5^Institute for Advanced Research in Chemical Sciences (IAdChem). Universidad Autónoma de Madrid. 28049 Madrid, Spain* |

**S1 Experimental section: Materials and Instrumentation**

**Materials**

All reagents and solvents were purchased from standard chemical suppliers: CuSO_4_·5H_2_O >98% Prolabo; CuSO_4_ anhydrous >99% Panreac; Cu(NO_3_)_2_·3H_2_O extra pure and sodium hydroxide (NaOH) Scharlau; Gelatin Bloon 100-120 Angel Jobal (animal origin); 3,3′,5,5′-Tetramethylbenzidine (TMB) Sigma Aldrich; MeOH 99.8% and EtOH 99% Scharlau, and used as received. Phosphate-buffered saline (PBS) 10 mM was prepared as described in standard protocols [1] and Isophthaloyl bis β-alanine (H_2_IBA) was synthesized as described in the literature [2]. All microorganisms were from American Type Culture Collection (ATCC), and LB -Agar medium was from DifcoTM. LB-Agar was prepared in distilled water (1 L) by dissolving agar (15 g) and LB broth (25 g) autoclaving at 15 psi, from 121-124 °C for 15 minutes.

**Instrumentation**

**Infrared (FT-IR) spectra** were recorded on a PerkinElmer 100 spectrophotometer using a PIKE Technologies MIRacle Single Reflection Horizontal ATR Accessory from 4000–500 cm^−1^.

**Elemental analysis** was performed on an elementary microanalyzer LECO CHNS-932. It works with controlled doses of O_2_ and a combustion temperature of 1000 ᵒC.

**Powder X-ray diffraction (PXRD)** was collected using a PANalytical X'Pert PRO MPD θ/2θ secondary monochromator and detector with fast X'Celerator, which was used for general assays. Theoretical X-ray powder diffraction patterns were calculated using Mercury Cambridge Structural Database (CSD) version 4.0.0 software from the Crystallographic Cambridge Database. The samples were analysed with scanning θ/2θ.

**Thermogravimetric analysis (TGA)** was performed on a TGA Q500 Thermobalance with an EGA (Evolved Gas Analysis) furnace and a quadrupole mass spectrometer Thermostat Pfeiffer from Tecnovac, to analyse gases, which are given off from the sample. The powder sample was analysed using a Pt sample holder and N_2_ flow as purge gas of 90 mL/min with a heating ramp from room temperature to 1000 ᵒC at 10 ᵒC/min.

**Field emission scanning electron microscopy (FESEM)** images were recorded on a FEI VERIOS 460 and JEOL JSM 7600F field emission scanning electron microscopes. SEM-EDX images and EDX spectra were recorded using a Hitachi S-3000N microscope with an ESED coupled to an INCAx-sight EDX analyzer. For this technique, the samples were metallized with a gold layer of 15 nm, under a pressure of 10^−3^ Pa. Bacteria samples for SEM were rinsed with PBS three times and centrifuged at 7000 rpm for 5 min. Then, they were fixed with 2.5 % glutaraldehyde for 3 h at 4 ºC. After that, bacteria were dehydrated in ethanol solutions with a graded series (20-100%) for 10 min each time and stored at 4 °C 48 h. Finally, they were deposited by drop-casting on a SiO_2_ surface.

**Atomic Force Microscopy (AFM)** images were acquired in dynamic mode using a Nanotec Electronica system operating at standard conditions. For AFM measurements, Olympus cantilevers were used with a nominal force constant of 0.75 N/m and a resonance frequency of about 70 kHz. The images were processed using WSxM. The surfaces used for AFM were SiO_2_ 300 nm thickness (IMS Company). SiO_2_ surfaces were sonicated in an ultrasound bath at 37 Khz and 380 Watts, for 15 min in acetone, 15 min in 2-propanol, and then dried under an Argon flow. 40 µL of the sample diluted in Milli-Q water were deposited on the SiO_2_ substrate by drop-casting, allowing to adsorb for 15 min at room temperature.

**Magnetic measurements** were done in a Quantum Design MPMS-XL-5 SQUID magnetometer in the 2-300 K temperature range with an applied magnetic field of 0.1 T to the crystalline samples.

Qualitative and quantitative **Total X-ray Fluorescence (TXRF)** analyses were performed with a benchtop S2 PICOFOX TXRF spectrometer from Bruker Nano (Germany). This is equipped with a Molybdenum X-ray source working at 50 kV and 600 µA, a multilayer monochromator with 80% of reflectivity at 17.5 keV (Mo K_α_), a XFlash SDD detector, with an effective area of 30 mm^2^, and an energy resolution better than 150 eV for 5.9 keV (Mn K_α_). The acquisition time for qualitative analysis was 300 s and for the quantitative analysis was 600 s. Titanium was chosen as the internal standard for quantification mainly because this element was not present in the samples and to avoid chemical distortion of the samples and volatilization of Cl and S. The Spectra 7 software from Bruker was used for control, acquisition, deconvolution, and integration of all analyzed samples.

**S2 Synthesis of 1, 1n and 1n@Gelatin**

**Synthesis of [Cu_2_(IBA)_2_(OH_2_)_4_]_n_·6nH_2_O (1):** A mixture of Cu(NO_3_)_2_ 3H_2_O (0.1 g, 0.4 mmol), H_2_IBA (0.127 g, 0.4 mmol) and NaOH (0.033 g, 0.8 mmol) was stirred for 1h at room temperature in 16 ml of MilliQ water (pH=3.4). After that, a blue solid was obtained, filtered off, washed with water, ethanol, and diethyl ether, and dried in air (compound **1**). After one week, light-blue needle crystals can also be obtained from the mother liquors in a Petri dish at room temperature. Its structure was previously published by S. Lymperopoulou et al. [2] “Fig. 1”. Yield 16 % based on Cu. Anal. Calcd. (found) % for C_28_H_48_Cu_2_N_6_O_22_: C, 36.56 (36.56); H, 5.26 (5.14); N, 6.09 (6.10). The IR (Figure S1) has the following characteristic bands (cm^-1^): 3387 (m), 3281 (s), 1659 (m), 1623 (s), 1563 (s), 1531 (s), 1407 (w), 1078 (w), 715 (s), 684 (s). PXRD of the solid has been performed and corresponds to compound **1** “Fig. S2”.

**Synthesis of [Cu_2_(IBA)_2_(OH_2_)_4_]_n_·6nH_2_O 1n@MOG and 1n***:* A solution of CuSO_4_ anhydrous (1.2 g, 7.5 mmol) in 5 ml of MilliQ water was rapidly added to another solution formed by H_2_IBA (1.54 g, 5 mmol) and NaOH (0.4 g, 10 mmol) in 11 ml of MilliQ water (pH=3) both at room temperature. Blue fibers were instantly observed. When the mixture was stirring for 5 min, in a sonication bath (37 GHz, 100 % powder), an unstable gel 1n@MOG was formed. This MOG can be transformed into a nano-crystals (1n) after still sonication in the same conditions for 20 min more. Yield 20 % based on Cu. Anal. Calcd. (found) % for C_28_H_48_Cu_2_N_6_O_22_: C, 36.56 (36.92); H, 5.26 (4.99); N, 6.09 (6.15). The IR has the following characteristic bands (cm^-1^) [3]: 3388 (m), 3286 (s), 1660 (m), 1628 (s), 1561 (s), 1531 (s), 1406 (w), 1080 (w), 720 (s), 684 (s) (Figure S1). PXRD of the solid has been performed and corresponds to compound 1 “Fig. S3” [4].

**Synthesis of 1n@Gelatin:** 0.21 g of gelatin was dissolved in 2 ml f of Milli-Q water at room temperature. Subsequently, 20.8 mg of 1n was added to the solution and stirring for 1 h. The mixture was left to rest at 4 ºC for 10 min until it got consistency. PXRD of the gelatinous material has been shown in Fig. S5 [4].

**S3 Characterization of 1, 1n and 1n@Gelatin**

**Figure S1** IR spectra of [Cu_2_(IBA)_2_(OH_2_)_4_]_n_·6nH_2_O (1) where black line represents 1 and green line 1n.

**Figure S2** PXRD patterns of [Cu_2_(IBA)_2_(OH_2_)_4_]_n_·6nH_2_O (1). Blue line corresponds to the experimental data and black line corresponds to simulated data. Peaks have been assigned using Mercury 2020.2.0 from ****CSD software package [4].

**Figure S3** PXRD patterns of [Cu_2_(IBA)_2_(OH_2_)_4_]_n_·6nH_2_O. Blue line corresponds to 1n and black line corresponds to 1. Peaks correspond to the ones shown in Fig S2.

**Figure S4** SEM images of compound 1 synthesized using stirring (1400 rpm) and Cu(NO_3_)_2_·3H_2_O (a) or (b) CuSO_4_ anhydrous (b) as starting salts.

**Figure S5** PXRD patterns of pristine compound 1 (black line), 1n@Gelatin (light blue line).

**S4 Solvent and thermal stability of 1n**

Compound **1n** is stable at room temperature in water at neutral pH, however it turns into white solid and dissolves at acid pHs (lower than 3) and degrades at basic pHs.

**Figure S6** (a) Stability of 1n in aqueous solution at pH 3 after 10 min (left) and 2 hours (right); (b) SEM image of 1n turned into white solid formed before its total dissolution.

**Figure S7** (a) Stability of 1n in aqueous solution at pH 9 after 10 min (left) and 2 hours (right); (b) SEM image of degraded solid (1n pH 9, after two hours).

The thermal stability of compound **1n** has been studied finding that, all water molecules were lost at 254 ºC in three stages. The six solvation molecules further two coordination molecules (obs. 14.97%; calc. 15.2%). The last remaining two water molecules were lost in two steps with a total weight loss of 3.5 % (cald 3.8%). After 254 ºC, H_2_IBA ligands were mainly lost as CO_2_ and water (obs. 60.2%; calc. 65%). The loss of fragments such as the aromatic ring (m/z=77) and part of the aliphatic chain (m/z=72) are also appreciated.

**Figure S8** Thermal stability of compound 1n. Thermogram signals: black and the red line (weight (TG) and derivate weight (DTG), represent the stages of the losses produced. Multicolor-labeled lines represent the ion current associated with each mass lost in each step.

Besides, compound **1n** presents thermo- and solvatochromic properties leading to a new green less crystalline phase when temperature up 100 ºC is applied “Fig. S9”. Compound **1n** can be recovered, from this less crystalline new phase, by immersing it in water “Fig. S10”.

**Figure S9** Color change of compound 1n, from blue to green, when it is heated at 100 ºC for 20 min (thermochromism). This green material returns to pristine 1n when it is immersed in water (solvatochromism).

**Figure S10** PXRD patterns of [Cu_2_(IBA)_2_(OH_2_)_4_]_n_·6nH_2_O. The blue line corresponds to 1n, the green line corresponds to 1n heated at 100 ºC, and the light-blue line to 1n immersed in water after being heated.

The thermal stability of pristine compound **1n** and **1n** previously heated at 100 ºC “Fig. S8, S11” has been done to check the number of lost water molecules that can cause its thermochromism. In the pristine compound, all water molecules were lost at 254 ºC in three stages (obs. 14.97%; calc. 15.2%) starting at 100 ºC with the loss of the first one. Once compound **1n** changed its color to green was again thermically analyzed to confirm that a lower weight-loss of water than the above thermogram was produced. In this thermogram, the initial stage had a loss of 6.5 % versus 15 %. This percentage could indicate the loss of three solvation water molecules, and the rest stages seem to be similar to the before one.

**Figure S11** Thermal stability of compound 1n after being previously heated at 100 ºC. Black and red lines represent the weight (TG) and derivate weight (DTG), respectively, showing the stages of the losses produced. Multicolor-labeled lines represent the ion current associated with each mass lost in each stage.

**S5 Study of magnetic properties**

The thermal variation of the product of the molar magnetic susceptibility per two Cu(II) ions times the temperature (ꭕ_m_T) shows a room temperature value of 0.80 cm^3^ K mol^-1^, which is the expected value for two independent S = ½ Cu(II) ions with g ≈ 2.1. When the sample is cooled, ꭕ_m_T remains constant down to around 15 K and shows an abrupt decrease at lower temperatures “Fig. S12(left)”. This behaviour indicates the presence of a weak antiferromagnetic coupling that is further confirmed by the presence of a maximum at low temperatures in the thermal variation of ꭕ_m_ (inset in Fig. S12(left)). Since the structure of **1n** shows the presence of a Cu(II) dimer with a double oxido bridge, we have fitted the magnetic properties of **1n** to a simple S = ½ Bleaney-Bowers dimer model. This model reproduces very satisfactorily the magnetic properties of **1n** with g = 2.1445, J = -6.0 cm^-1^ and a monomeric paramagnetic impurity of 3.1 % (the Hamiltonian is written as H = -JS_1_S_2_).

The isothermal magnetization at 2 K shows a linear increase for fields below *ca*. 2 T with an increase in the slope for fields above 3 T and an inflexion point at around 5.5 T “Figure S13(left)”. The inflexion point can be clearly seen as a maximum in the plot of the derivative of the magnetization with the magnetic field “Figure S13(right)”. This behaviour suggests that compound **1n** is a metamagnet with a critical field of ca. 5.5 T at 2 K. Below this critical field, **1n** presents an antiferromagnetic coupling, whereas for fields above the critical field it shows a ferromagnetic coupling. At 5 K **1n** shows an antiferromagnetic coupling with no metamagnetic transition for fields below 7 T “Fig. S13”.

A further confirmation of the metamagnetic behavior is provided by the measurements performed with different magnetic fields “Fig. S12(right)”. Thus, these measurements show a maximum in the ꭕ_m_ *vs*. temperature plot at low temperatures that disappears for magnetic fields above *ca*. 5.5 T.


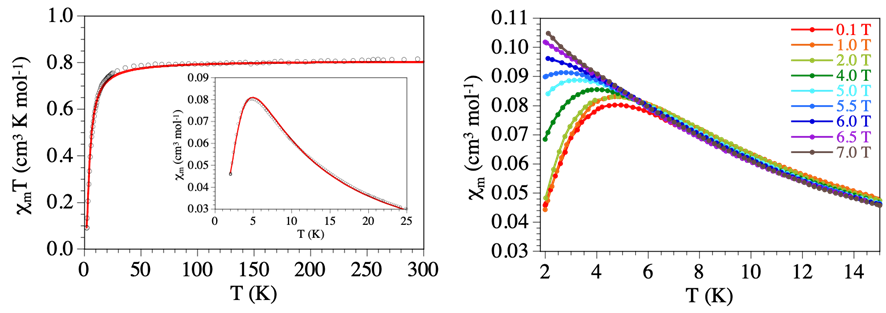


**Figure S12** (left) 1n, thermal variation of χ_m_T. Inset shows the low temperature region of the c_m_ *vs.* temperature plot). Solid lines are the fit to a S = ½ dimer model (see text). (right) 1n, thermal variation of χ_m_ with different applied fields in the 2-15 K range, showing the disparition of the maximum for fields above *ca*. 5.5 T.

**
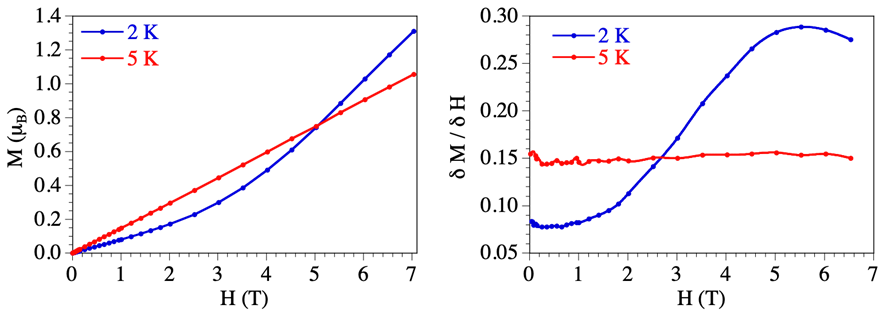
**

**Figure S13** (left) Isothermal magnetization at 2 and 5 K for compound 1n. (right) Variation of the derivative of the magnetization with the magnetic field for 1n.

**S6 Catalytic activity: Methods**

**Catalase-like activity measurement**

Catalase-like activity of 1n was assessed spectrophotometrically by a reaction with potassium dichromate/acetic acid method [5]. In this method, in the presence of H_2_O_2_, dichromate in acetic acid is reduced to chromic acetate, which can be evaluated spectrophotometrically at 570 nm. The change in absorbance was measured against the reagent blank.

Enzyme activity was investigated by the incubation of 3 mg of 1n in 100 µL of PB buffer (50 mM, pH 7.4) containing 0.13 M of H_2_O_2_ for 2.5 hours at room temperature. After incubation, 200 µL of 5 mL of a 5 % aqueous solution of potassium dichromate in 15 mL of glacial acetic acid was added. The samples were kept at 100°C for 10 minutes and then cooled with tap water. As a control test sample, all reagents were mixed except for compound 1n, which was replaced by the same volume of PB buffer. Data corresponded to mean values ± standard deviation from four different experiments. For statistical calculations, one-way ANOVA and Tukey’s Test and R Commander Software were used. P-value < 0.001 (***) was considered as statistically significant.

**Peroxidase-like activity measurement**

The catalytic activity of the 1n compound (3 mg/mL) in the presence of H_2_O_2_ (0.05 mL) as a peroxidase enzyme was investigated through the oxidation of tetramethylbenzidine (TMB) (0.05 mL, 10 mM). The experiments were carried out in phosphate-buffered saline (PBS) (pH 7.5, 50 mM) and monitored spectrophotometrically (JASCO V-550 UV/vis spectrometer) from 240 nm to 700 nm and the activity at 440 nm for 10 minutes.

**SOD-like activity measurement**

The SOD activity of the 1n was evaluated employing the nitroblue tetrazolium (NBT) method [6], using xanthine/xanthine oxidase as a source of superoxide anion. The superoxide anion promotes the reduction of NBT to formazan, which can be evaluated spectrophotometrically at 560 nm.

All stock solutions were made fresh on the day of use. Xanthine solution (50mM) was prepared in PB buffer (50mM, pH 8) containing 0.3 M of sodium hydroxide. NBT (2.3 mM) was prepared in PB buffer (50mM, pH 8). The reaction medium had PB buffer (50mM, pH 8), 3mM xanthine, 0.02 U/mL xanthine oxidase, 25.5 µM NBT and the test sample at different concentrations in a total volume of 100 µL. A control experiment was conducted by mixing all reagents, except that the test sample was substituted with an equal volume of PB buffer.

The change in absorption was monitored over 30 minutes “Fig. S14, S15”. The absorption reached a maximum after 20 minutes of reaction and was used to calculate the IC_50_. The experiments were done in triplicates, and the IC_50_ was calculated from a linear regression analysis (See “Fig. 7”).

**Figure S14** Absorbance measurement against reaction time when the absorption reaches a maximum.

**Figure S15** Measurements of NBT absorption spectra at 560 nm upon 20 min of reaction to different concentrations of 1n.

**S7 Copper release and antibacterial experiments**

**Sample preparation for copper release assays**

0.05 g of compound 1n was dispersed in 5 ml of deionized water. The sample's dispersion was left shaking at 140 rpm and room temperature. After 24 h, the sample was centrifuged in a High-Speed Brushless Centrifuge MPW-350R for 2 h at 2500 rpm. The supernatant was filtered and analyzed. Then the medium was replenished with another 5 ml of freshwater, and the sample was again shaken. This procedure was repeated until seven days (t=1, 3, 6, and 7 days). Supernatant from single-water samples was analyzed by TXRF “Fig. S16”.

**Figure S16** Accumulative copper concentration of compound 1n in water after carrying out copper release assays.

**1n** antibacterial activity was tested by agar diffusion against different microorganisms (*E. coli DH5*α, *E. coli Bl21*, *E. coli K12*, *E. coli XL1Blue*, *P. fluorescens*, *S. spiritovirum*, *B. cereus*, *B. circulans*, *B. subtilis*, *S. epidermis*, *A. faecalis*, *D. radiodurans* and *L. lactis*). Briefly, a standardized inoculum of the microorganism is swabbed onto the surface of LB-agar plate. Normally, filter paper disks are impregnated with a standardized concentration of an antimicrobial agent placed on the surface. The size of the zone of inhibition around the disk is measured after overnight incubation at 37ºC. In our case, after swabbed of the microorganism, we have directly added the antimicrobial agent or compounds onto the plate, so the diameter of the inhibition zone properly describes the antimicrobial potency of the compounds. We have used ampicillin (2 µL at 3 mg/mL), kanamycin (2 µL at 3 mg/mL), **1n** (1-20 µL at 3 mg/mL), **1n**:H_2_O_2_ (1-20 µL:1 µL) as antimicrobial agents and CuSO_4_ (20 µL at 3 mg/mL) or H_2_O_2_ as control.

The antimicrobial activities of **1n**, and in the presence or absence of H_2_O_2_, the minimal inhibitory concentration (MIC), and the zone of inhibition against *Sphingobacterium* or *E. coli* were studied by using optical density at 600nm and the colony count method [7].

To determinate the growth curve of bacteria, the compound **1n** was dispersed at different concentrations (0.02-0.16 mg/ml) into sterilized tubes with liquid broth medium (LB) and 50μL of H_2_O_2_ 25mM in water “Fig. S18”. Briefly, 50μL of well-cultivated bacterial solution of *Sphingobacterium* (10^6^ CFU/mL, overnight) was added to each tube with 5 mL of LB and different concentrations of **1n** compound plus H_2_O_2_ (25 mM) and were left 24 h to shake at 37 ºC. Absorbance measurements of the samples were recorded at 600 nm each hour. In the case of MIC assay, when the OD_600nm_ reached 0.5 value, was added the **1n** at different concentrations, and H_2_O_2_ (25 mM).

The minimal inhibitory concentration (MIC) of the compound **1n** without and with H_2_O_2_ was assayed “Table S1”. Briefly, 25 μL of well-cultivated bacterial solution of *Sphingobacterium* (10^6^ CFU/mL) was added to 5 mL of M-H with shaking at 37 ºC until it reaches a OD_600nm_= 0.5. Then, **1n** alone at different concentrations (control) or with 50 μL of H_2_O_2_ at 25 mM were added. The absorbance was tested with the time at 600nm. The 25 μL of supernatant of two samples (*Sphingobacterium* broth and the same broth with 0.16 mg/ml of **1n** plus 50 μL H_2_O_2_ (25 mM)) was extracted from each tube after 24 h and was uniformly spread over agar nutrient plates.

**Figure S17** Effect of different concentrations of H_2_O_2_ (12.5, 25, 100, 300 mM) on a *Sphingobacterium* culture dish.

**Table S1** Strains tested against compound 1n and its minimum inhibitory concentration (MIC).

|  | **MIC (μg/ml)** | | | |
| --- | --- | --- | --- | --- |
| ***Strains/*Gram (+/-)** | **H_2_IBA ligand** | **1n** | **1n + H_2_O_2_** | **Amp** |
| ***Escherichia coli DH5a* (-)** | >3 | >3 | >3 | <0.3 |
| ***Escherichia coli Bl21*(-)** | >3 | >3 | >3 | <0.3 |
| ***Escherichia coli K12*(-)** | >3 | >3 | >3 | <0.3 |
| ***Escherichia coli XL1Blue*(-)** | >3 | >3 | >3 | <0.3 |
| ***Pseudomonas aeruginosa* (-)** | >3 | >3 | >3 | <0.3 |
| ***Pseudomonas fluorescens*(-)** | >3 | >3 | >3 | <0.3 |
| ***Sphingobacterium* *spiritovirum*(-)** | >3 | >3 | <0.1 | >1 |
| ***Bacillus cereus*(+)** | >3 | <3 | <1 | <0.3 |
| ***Bacillus circulans* (+)** | >3 | >3 | >3 | <0.3 |
| ***Bacillus subtilis* (+)** | >3 | >3 | >3 | <0.3 |
| ***Staphylococcus epidermis*** | >3 | >3 | >3 | <0.3 |
| ***Alcaligenes faecalis* (-)** | >3 | >3 | <1 | >1 |
| ***Deinococcus radiodurans* (+)** | >3 | >3 | >3 | <0.3 |
| ***Lactococcus lactis* (+)** | >3 | >3 | >3 | <0.3 |

**Figure S18** (a) Growth curves of *Sphingobacterium* in different concentrations of compound 1n (20 μg/ml-blue, 40 μg/ml-green, 60 μg/ml-purple and 160 μg/ml-yellow with H_2_O_2_ (25mM). Blank with only *Sphingobacterium* (Blackline) and control *Sphingobacterium* with only 1n compound (Redline). (b) MIC curves of *Sphingobacterium*, at OD_600nm_=0.5 different concentrations of 1n compound were added (20 μg/ml-blue, 40 μg/ml-green, 60 μg/ml-purple and 160 μg/ml-yellow) with H_2_O_2_ (25mM). Blank with only *Sphingobacterium* (black line) and red line *Sphingobacterium* with only 1n compound. (c) agar-plates of *Sphingobacterium* strain (supernatant) after 24h in M-H broth (top) and the presence of 160 μg/ml of 1n compound with H_2_O_2_ (bottom).

**Bacterial reduction assay using 1n@Gelatin**

0.15 mL of a well-cultivated bacterial solution of *Sphingobacterium* spiritovirum or *E. coli DH5α* (10-8 dilution of overnight medium) was uniformly spread well over solid ager nutrient plates.Then, 0.15 ml of H_2_O_2_ at 25 mM were added as blank and 0.4mg of 1n@Gelatin plus H_2_O_2_ at the same conditions were adaded as sample. The colonies were counted after incubation at 37 ºC for 24 h for the blank and for the 1n@Gelatin and the percentage of inhibition of bacterial growth was evaluated using the following equation:

K inhibition (%) = (N_blank_-N_sample_)/N_blank_ * 100

where K is the percentage of inhibition, Nblank is colony forming units (CFU) of bacterium in presence of H_2_O_2_ (0.15ml at 25mM) and Nsample is CFU of viable colonies adding 0.4mg of 1n@Gelatin plus peroxide of hydrogen.

**S8 References**

[1] Standard Operating Procedures (SOPs) Laboratorio de Genómica Viral y Humana Facultad de Medicina UASLP, 2008.

[2] Lymperopoulou, S.; Dokorou, V. N.; Tsipis, A. C.; Weidler, P. G.; Plakatouras, J. C.; Powell, A. K.; Kostakis, G. E., Influence of the metal salt on the self-assembly of isophthaloylbis-β-alanine and Cu(II) ion. *Polyhedron* **2015,** *89*, 313-321.

[3] Pretsch, P.; Bühlmann, P.; Badertscher, M. Structure Determination of Organic Compounds. Tables of Spectral Data. 4^th^ edition published by Springer, Berlin, 2009.

[4] Macrae, C. F.; Edgington, P. R.; McCabe, P.; Pidcock, E.; Shields, G. P.; Taylor, R.; Towler, M.; van de Sreek, J. Mercury: visualization and analysis of crystal structures. *J. Appl. Cryst.* **2006**, 39, 453-457.

[5] Kubota, R.; Asayama, S.; Kawakami, H., A bioinspired polymer-bound Mn-porphyrin as an artificial active center of catalase. *Chem. Commun.* **2014,** *50* (100), 15909-15912.

[6] Ward, M. B.; Scheitler, A.; Yu, M.; Senft, L.; Zillmann, A. S.; Gorden, J. D.; Schwartz, D. D.; Ivanović-Burmazović, I.; Goldsmith, C. R., Superoxide dismutase activity enabled by a redox-active ligand rather than metal. *Nature Chem.* **2018,** *10* (12), 1207-1212.

[7] Balouiri, M.; Sadiki, M.; Ibnsouda, S. K., Methods for in vitro evaluating antimicrobial activity: A review. *J. Pharm. Anal.* **2016,** *6* (2), 71-79.
